# Feasibility Study of Bioactive Hydrogel Coatings on Ti-6Al‑4V Gyroid Scaffolds for Bone Tissue Engineering

**DOI:** 10.1021/acsbiomaterials.4c02250

**Published:** 2025-05-30

**Authors:** Lisa Schöbel, Maddi Garcia Ayerbe, Christian Polley, Gurutze Arruebarrena, Hermann Seitz, Aldo R. Boccaccini

**Affiliations:** † Institute of Biomaterials, Department of Materials Science and Engineering, 9171 Friedrich Alexander-University Erlangen-Nuremberg, 91056 Erlangen, Germany; ‡ Faculty of Engineering, 16630 Mondragon University, 20500 Arrasate/Mondragon, Spain; § Chair of Microfluidics, Faculty of Mechanical Engineering and Marine Technology, 249089 University of Rostock, 18059 Rostock, Germany; □ Department Life, Light Matter, University of Rostock, 18059 Rostock, Germany

**Keywords:** Bioactive Hydrogels, Triple Periodic Minimal Surface, Hard-to-Soft Transition

## Abstract

Titanium alloys are commonly used for bone replacement due to their excellent corrosion resistance. However, they can cause stress shielding due to their high stiffness. Consequently, porous titanium scaffolds can be designed to reduce the elastic mismatch with bone, and further hydrogel coatings can be applied to mimic the extracellular microenvironment. In this work, gyroid titanium scaffolds were coated with a bioactive alginate-gelatin hydrogel. The addition of 45S5 bioactive glass enhanced the mechanical properties of the hydrogel and its adhesion strength. Furthermore, the developed hydrogels allowed for the penetration of gyroid scaffolds, demonstrating the potential of bioactive coatings for titanium implants.

## Graphical abstract

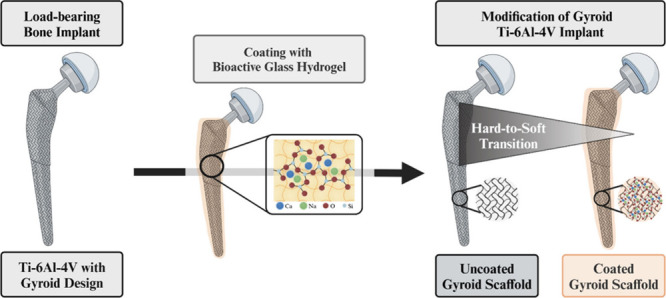

## Introduction

Bone defects are caused by various factors, such as lack of physical activity, surgical procedures, or the natural aging process, which can lead to osteoporosis.^1^ While bone tissue typically has strong regenerative potential, bone defects beyond a critical size cannot heal without external support, which has driven research in bone tissue engineering (BTE).^2^ Current treatments for critical size defects include autogenous grafts, allogenic grafts, xenografts, and alloplastic materials.^3^ Metals, such as stainless steel, Co–Cr alloys, and titanium-based alloys, are vital alloplastic materials in load-bearing applications.^4^ Titanium alloys, particularly Ti-6Al-4V, are widely used for bone replacement due to their biocompatibility and high corrosion resistance.^4^ However, Ti-6Al-4V has some drawbacks, including the mechanical mismatch with natural bone, which can cause stress-shielding and implant loosening.^5^ To address this, porous scaffold designs based on triple periodic minimal surface (TPMS) structures have gained attention due to their biomimicry and excellent porosity-to-strength ratio.^6^ In previous work, we demonstrated that the mechanical properties can be controlled by varying the unit cell size of electron beam melt manufactured scaffolds.^7^

Another approach to improve implant integration involves tailoring the interface between scaffold and bone by introducing a softer material as a coating, such as hydrogels.^8–10^ Several studies have reported a positive influence of locally softer surfaces on cell integration.^8^ Hydrogels, such as those based on alginate dialdehyde (ADA) and gelatin (GEL), resemble the extracellular matrix and show tailorable properties, e.g., with regard to mechanical properties and degradation behavior.^9,10^ Thus, ADA-GEL is a highly researched hydrogel for biofabrication and drug delivery, and its suitability for BTE was also reported.^10^ Moreover, bioactive glasses (BGs), such as 45S5 BG, can be added to introduce bioactivity. Consequently, ADA-GEL-BG composite hydrogels are promising candidates for engineering soft coatings on titanium alloy scaffolds that mimic the extracellular matrix microenvironment aiming to overcome the bioinertness of uncoated titanium scaffolds. To the best of the authors’ knowledge, the coating of gyroid Ti-6Al-4V scaffolds with hydrogels is a new field of research with only a few publications on the subject,^11,12^ thus representing a novel strategy to functionalize porous titanium implants. Although, it is well-known that the pore size has a detrimental effect on the mechanical performance and bone-implant interactions, this study builds on our previous work on gyroid scaffolds by using the same pore sizes^7^ and focusing on the feasibility of coating such gyroids with alginate-gelatin-based hydrogels enriched with bioactive glass.

## Results and Discussion

Because of the onset of gelation of alginate-based hydrogels by the release of divalent ions from incorporated 45S5 BG,^13^ both suitable hydrogel formulations and the highest possible BG concentration must be identified. A tube inversion test determined the gelation time for 2.5/5.0% ADA-GEL with different BG concentrations (Figure S1A,B). For the dip-coating procedure, a gelation time of 5 min was defined as the minimum time for the coating to be feasible. As shown in Figure S1B, BG concentrations lower than 0.25 wt % were considered suitable. However, hydrogels with 0.25 wt % showed the formation of highly cross-linked hydrogel clumps, which rendered them not useable for coating porous scaffolds. Consequently, 0.1 wt % BG and an intermediate concentration of 0.05 wt % BG were chosen. Figure 1 shows the appearance of hydrogel-coated scaffolds cut open by spark erosion (see also Figure S2 for visual inspection of coated gyroids from the outside). The hydrogel was identified inside the pores of gyroid scaffolds with large and medium pore sizes for all investigated hydrogel formulations. An exception was made by the smallest pore size (0.84 mm, Table S1), where none of the hydrogel formulations penetrated the inside of the scaffolds. The effect of porosity on the feasibility of hydrogel coatings was investigated by determining the weight gain after the coating procedure (Figure S3). As expected, scaffolds with higher porosity allowed for more hydrogel absorption due to the increased available space and reduced capillary effects. The inherent viscosity of the ADA-GEL-based hydrogel limits the pore penetration ability, making a careful selection of pore size crucial. While reducing the hydrogel concentration can lower viscosity and improve pore penetration, it results in softer hydrogels that degrade more rapidly. Nevertheless, it can be concluded that the penetration of highly porous Ti-6Al-4V scaffolds with the proposed ADA-GEL-BG hydrogels is feasible after carefully selecting the hydrogel formulation and pore size of the scaffold.

**Figure 1:**
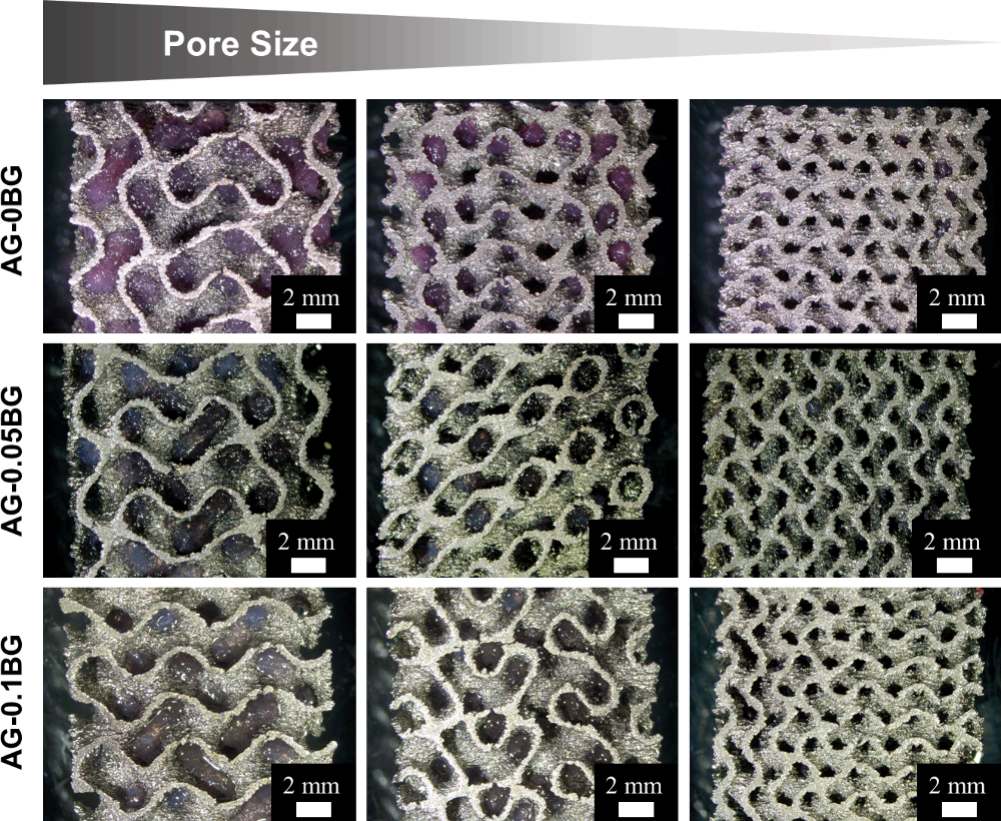
Light microscopy images of ADA-GEL-BG-coated gyroid scaffolds after spark erosion to evaluate the hydrogels’ ability to penetrate different pore sizes.

The swelling and degradation behavior of ADA-GEL-BG hydrogels was studied over an incubation period of 3 weeks (Figure 2A). The bioactive glass-containing hydrogels exhibited higher swelling after 1 day compared to the pristine hydrogel, likely due to alterations in cross-linking dynamics and thus a looser polymeric network with increased pore volume, and the formation of hydrophilic silica layers that enhance water uptake. Thereafter, all hydrogels started to degrade with steadily decreasing weight until 21 days. However, the BG-containing hydrogels showed a slightly slower degradation caused by the additional ionic cross-linking of the hydrogel by released Ca^2+^ ions from the 45S5 BG as described previously.^14^

**Figure 2:**
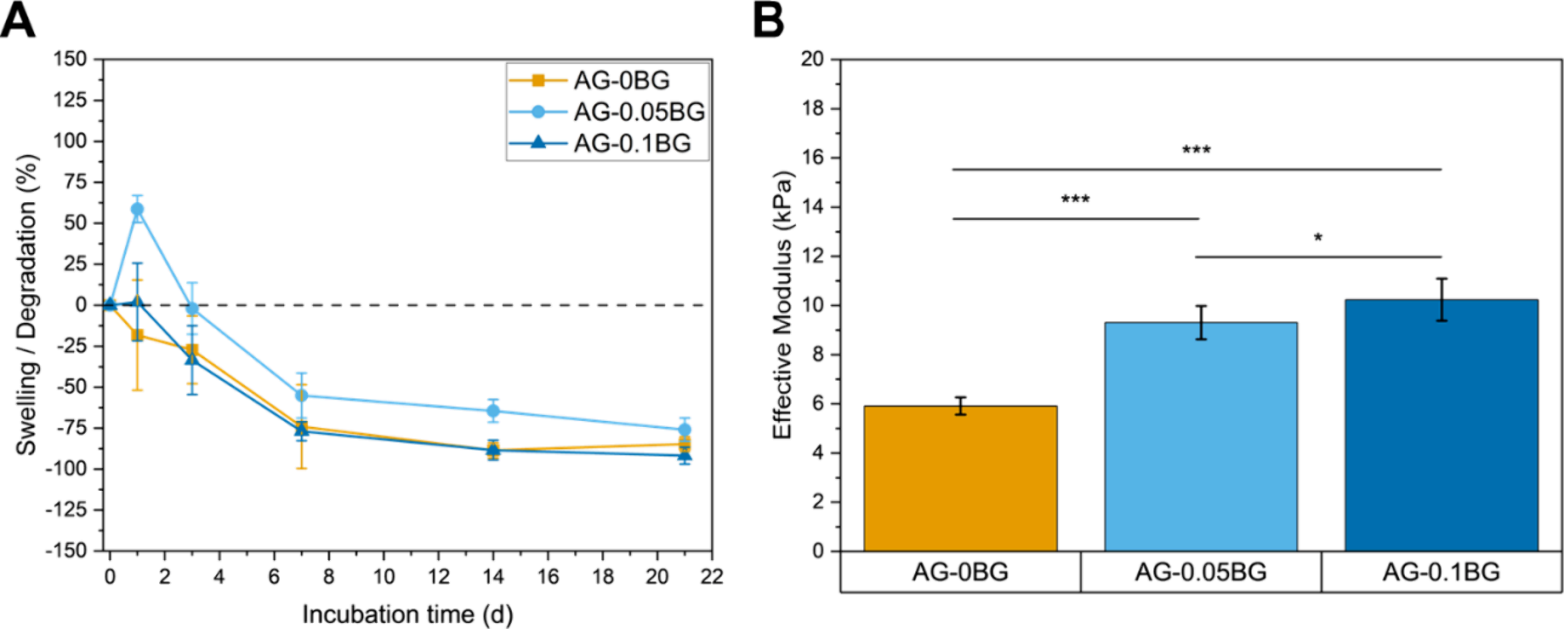
(A) Swelling and degradation behavior of hydrogels. (B) Compressive effective modulus of ADA-GEL-BG hydrogels.

The incorporation of bioactive glass into the coating served two purposes. On the one hand, BG was added to introduce bioactivity, which was studied by immersing hydrogel films into simulated body fluid at 37 °C for 2 weeks. After this incubation period, the typical bioactive behavior of BG-containing hydrogels was proven by the formation of hydroxyapatite, which was confirmed by X-ray diffraction analysis (Figure S4A). Moreover, cauliflower-like calcium-phosphate agglomerates were observed in scanning electron microscopy images (Figure S4B–D). On the other hand, the concentration of BG in the hydrogel formulation was varied to tailor the compressive modulus of the coating, which is represented in Figure 2B. As can be seen, the effective modulus ranged between 6–10 kPa, which is in a similar range as reported previously for BG-containing ADA-GEL.^14^ The introduction of bioactive glass increased the effective modulus of the hydrogel with increasing values for higher BG concentration. This stabilizing effect can be traced back to the additional ionic cross-linking of ADA by released Ca^2+^ ions^14^ and an enhanced bonding of ADA and GEL facilitated by the Si­(OH)_4_ groups of BG.^15^ Consequently, the mechanical properties of the coating are tailorable by varying the BG concentration. Moreover, the effective modulus could be tailored by changing the concentration of the used cross-linking agents^16^ or by employing longer cross-linking times.^17^ Another interesting approach to design the scaffold-bone interface could be realized by a multilayer coating employing ADA-GEL-BG coatings with different BG concentrations in each layer, creating a graded coating.

The adhesion of ADA-GEL-BG hydrogel coatings on the metallic substrate was tested by the pull-off adhesion test and the cross-hatch method (Illustration in Figure 3A). Figure 3B presents the results of the pull-off adhesion test, showing a slight increase in adhesion strength needed to remove ADA-GEL-0.1BG, though not statistically significant. However, this testing method is more suitable for thicker coatings, which is why this method has a constrained applicability for our hydrogel coatings with a thickness of approximately 200 μm (data not shown). Nevertheless, a complementary cross-hatch test indicated that the adhesion of BG-containing ADA-GEL to Ti-6Al-4V improved compared to pristine ADA-GEL, with the highest BG concentration performing best (Figure 3C). The appearance of the investigated samples was classified according to ASTM D3359-23 with 0B, 1B, and 2B for AG-0BG, AG-0.05BG, and AG-0.1BG, respectively. The improved adhesion of BG-containing ADA-GEL hydrogels could be explained by an improved chemical bonding to the titanium substrate, possibly due to silica and calcium oxides in BG.^18^

**Figure 3:**
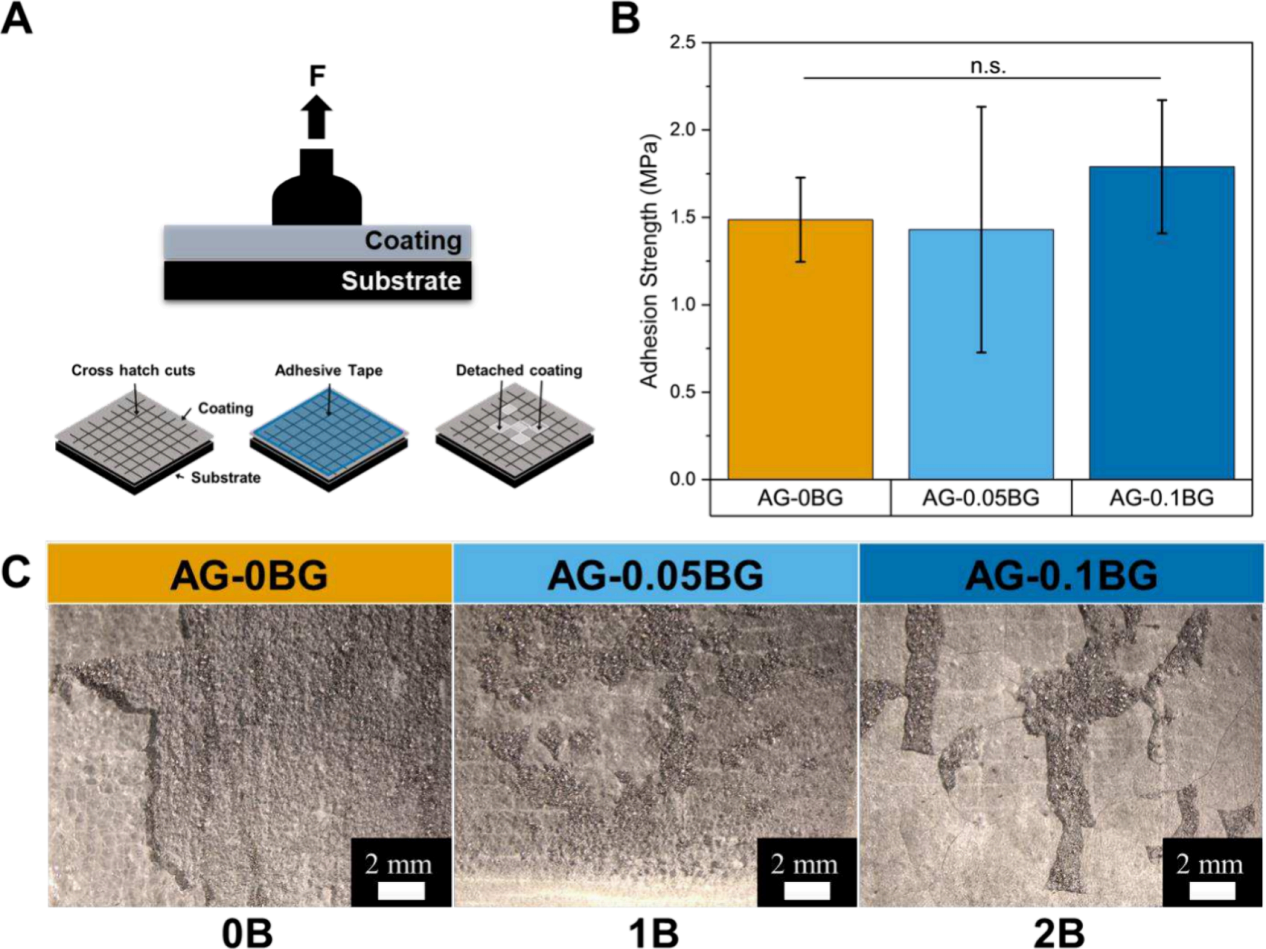
(A) Schematic illustration of pull-off and cross-hatch adhesion testing. (B) Results from pull-off adhesion test. (C) Results from cross-hatch test.

Finally, the cell-material interactions were studied by seeding MG-63 osteoblast-like cells on uncoated and ADA-GEL-BG-coated gyroid scaffolds. As shown in Figure 4 A, the metabolic activity of seeded MG-63 cells increased over the incubation period from 1 to 7 days with no significant difference between the material groups. However, after 7 days, a slight upward trend for hydrogel-coated Ti-6Al-4V gyroids can be distinguished, with BG-containing ADA-GEL showing slightly increased absorption values. Besides the proliferation of seeded cells, the cell morphology was also investigated by fluorescence microscopy of Phalloidin-DAPI stained cells. Figure 4 B shows that all materials are homogeneously covered by cells, demonstrating elongated and interconnected F-actin filaments. The cytocompatibility of ADA-GEL-BG hydrogels toward osteoblast cells in two-dimensional (seeding of cells) and three-dimensional (embedding of cells) cell culture has been shown extensively.^13,15^ Incorporating cells into the hydrogel with subsequent coating of the gyroid scaffolds represents another interesting approach that will allow for the loading of cells within the pores of the scaffold.

**Figure 4:**
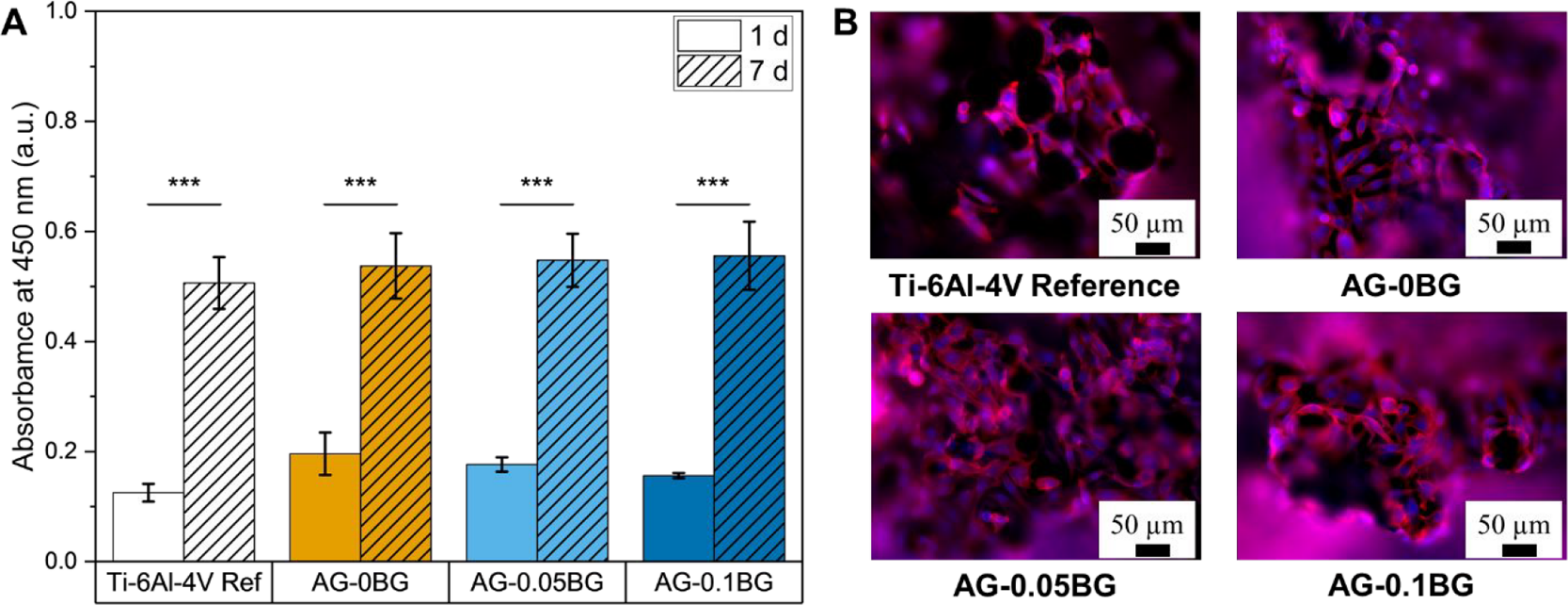
(A) WST-8 assay results from seeded MG-63 cells. (B) Fluorescence microscopy images of Phalloidin-DAPI stained MG-63 cells proliferating on uncoated and coated gyroid scaffolds after 7 days.

## Conclusions

In summary, coating Ti-6Al-4V gyroid scaffolds of various pore sizes with a bioactive, biocompatible, and biodegradable ADA-GEL-bioactive glass composite hydrogel was feasible. The addition of bioactive glass to the hydrogel-based coatings led to an increase in mechanical properties from 6 to 10 kPa and furthermore improved the adhesion to the titanium substrate determined by the cross-hatch method with 0B, 1B, and 2B for AG-0BG, AG-0.05BG, and AG-0.1BG, respectively. Consequently, the presented composite hydrogel coatings represent a versatile material platform that can be further exploited to, e.g., produce multilayer coatings of ADA-GEL hydrogels with graded stiffness or the delivery of cells into the pores of the gyroid scaffolds by embedding cells into the hydrogel.

## Supplementary Material



## Notes

Complete materials and methods along with supporting tables on the pore size of used gyroid scaffolds and hydrogel formulations as well as supporting figures on the gelation time determined by tube inversion test, light microscopy images of coated gyroid scaffolds, weight gain after coating and bioactivity results (PDF)
