# Supplementary material for: Feasibility Study of Bioactive Hydrogel Coatings on Ti-6Al‑4V Gyroid Scaffolds for Bone Tissue Engineering
Source: ACS Biomater Sci Eng. 2025 May 30;11(7):4057–61. doi: 10.1021/acsbiomaterials.4c02250 (PMC12264767; doi:10.1021/acsbiomaterials.4c02250)
Supplement: Supplementary file 1 [file ab4c02250_si_001.pdf]

Supporting Information

Feasibility Study of Bioactive Hydrogel Coatings on Ti-6Al-4V Gyroid Scaffolds for Bone Tissue Engineering

Lisa Schöbel[1], Maddi Garcia Ayerbe[2], Christian Polley[3], Gurutze Arruebarrena[2], Hermann Seitz[3], Aldo R. Boccaccini[1]

[1] Institute of Biomaterials, Department of Materials Science and Engineering, Friedrich-Alexander-University Erlangen-Nuremberg, 91056 Erlangen, Germany

[2] Faculty of Engineering, Mondragon University, 20500 Arrasate/Mondragon, Spain

[3] Chair of Microfluidics, Faculty of Mechanical Engineering and Marine Technology, University of Rostock, 18059 Rostock, Germany

Experimental Section

1. Materials

Alginate (Laminaria hyperborean, VIVA Pharm, PH176) was purchased from JRS PHARMA GmbH & Co. KG, Germany. The dialysis tubes (MWCO: 6-8 kDa) were obtained from Carl Roth GmbH + Co. KG, Germany. Dulbecco's Phosphate Buffered Saline (DPBS, [-] $CaCl_2$, [-] $MgCl_2$), Hank's Balanced Salt Solution (HBSS, [+] $CaCl_2$, [+] $MgCl_2$), Dulbecco's Modified Eagle Medium (DMEM, 1g/L D-glucose), Rhodamine-Phalloidin and DAPI were supplied by Thermo Fisher, Germany. The microbial transglutaminase (mTG, ACTIVA WM, specific enzyme activity of 85 − 135 U g^{-1}) was obtained from Ajinomoto Co., Inc., Japan. All other chemicals were purchased from Sigma Aldrich (Germany) if not stated otherwise.

2. Fabrication of Ti-6Al-4V gyroid scaffolds

For the different coating characterization techniques various titanium specimens, namely titanium discs (d = 10 mm, h = 3 mm), plates (20 x 20 x 3 mm^3) and gyroids with varying pore sizes (see Table S1) have been fabricated via electron beam melting (EBM) as presented by Polley *et al.*[1]

Table S1. Fabricated Ti-6Al-4V gyroids with pore size and porosity as reported by Polley et al.[1]

Label	As built Pore Size	As built Porosity
UC3	0.84 mm	71.01 %
UC4	1.15 mm	77.83 %
UC5	1.41 mm	81.71 %

3. Oxidation of alginate

The oxidation of alginate was performed following the protocol by Karakaya *et al.*[2] To summarize, 10 g of alginate derived from Laminaria hyperborean (VIVA Pharm, PH176, JRS PHARMA GmbH & Co. KG, Germany) were initially dispersed in 50 mL of ethanol, followed by the addition of an aqueous solution containing 9.375 mmol of $NaIO_4$. The resulting mixture was then stirred for a duration of 6 hours at room temperature (RT) in the absence of light. Subsequently, the reaction was quenched by introducing 10 mL of ethylene glycol and further stirring for 30 minutes. Following this step, the magnetic stirrers were turned off, allowing the reaction product to settle for 10 minutes after which the ethanol phase was decanted. The oxidized alginate (referred to as ADA) was then diluted with ultra-pure water (UPW) and transferred into dialysis tubes for dialysis against UPW with daily water changes. Finally, after 3 days of dialysis, the resulting ADA was frozen at -21°C for 24 hours and subsequently subjected to freeze-drying using a freeze dryer (LD1-2 Plus, Martin Christ GmbH, Germany).

4. Fabrication of hydrogels

All needed hydrogel components were firstly prepared as stock solutions. ADA was dissolved in DPBS under stirring overnight at room temperature (RT) to yield a hydrogel with a concentration of 7.5 w/v%. Furthermore, 10 w/v% gelatin (Type A, Bloom 300) was dissolved in UPW at 37°C under stirring for 30 min. Moreover, a dispersion of 45S5 Bioglass particles (BG, mean particle size of 2 µm, nominal composition in wt%: 45% SiO_2, 24.5% CaO, 24.5% Na_2O, 6% P_2O_5, Vitryxx®, Schott Aktiengesellschaft, Germany) in UPW was prepared. Finally, the hydrogel components were mixed according to the volume ratios shown in Table S2 and stirred for 10 min at 37°C similar to the published protocol of Reakasame *et al.*[3] Depending on the experimental setup, the hydrogel was either directly used for coating experiments or hydrogel films were produced by transferring 0.2 mL of the respective hydrogel formulation to a custom made silicone mold (d = 10 mm, h = 2 mm). Subsequently, the films were stored for 10 min at 4°C to facilitate the thermal gelation of gelatin and finally a dual ionic and enzymatic crosslinking was performed by immersing the hydrogels in a crosslinking solution for 10 min composed of 0.25 M $CaCl_2$ and 2.5 w/v% microbial transglutaminase. For the production of sterile hydrogels needed for swelling and degradation studies, investigation of bioactivity as well as *in vitro* studies, the hydrogels were produced in a sterile environment using sterile filtrated ADA as well as GEL stock solutions and BG was heat-treated at 160°C for 2 h and dispersed in sterile UPW. The crosslinking solution was also sterile filtrated and all other needed utensils were autoclaved.

Table S2. Volume ratio of all hydrogel components for the preparation of ADA-GEL-BG hydrogels

Label	7.5 w/v% ADA	10 w/v% GEL	UPW	0.3 w/v% BG	0.6 w/v% BG
AG-0BG	2	3	1	-	-
AG-0.05BG	2	3	-	1	-
AG-0.1BG	2	3	-	-	1

5. Dip-coating procedure

For the coating of Ti-6Al-4V gyroid scaffolds or plates, the hydrogel precursor solutions were prepared and after stirring the solutions for 10 min at 37°C, the stirring was turned off, and the gyroid scaffolds were dipped into the hydrogel solution. Finally, the scaffolds were removed from the solution, stored at 4°C for 10 min and crosslinked as described before.

6. Investigation of hydrogel properties

Gelation time

The gelation time was determined by employing the tube inversion test as described by Nguyen and Lee[4]. Here, 1 mL of the hydrogel precursor solution was transferred to a glass beaker (d = 25 mm) and the beaker was kept at room temperature while flipping it every 1 minute. The gelation time is determined as the elapsed time when no flowing was observed. Each hydrogel composition was tested with three replicates.

Swelling and degradation

For the investigation of the swelling and degradation behavior, the hydrogel films were prepared under sterile conditions. Subsequently, the films were incubated in 5 mL DMEM and stored in an incubator set to 37°C, 5% CO_2 and 95% humidity. At specific time points the hydrogel specimens were removed from the incubator and weighed again. The swelling or degradation behavior was determined using the following equation:

$$Swelling\ or\ Degradation\ (\%) = \frac{m - m_0}{m_0} \cdot 100\%$$

Where m represents the weight of the sample at the specified time point, and m_0 represents the weight of the film before immersion in medium. The DMEM was exchanged three times a week to imitate cell culture conditions. This study was performed with six replicates (n = 6).

Effective stiffness of hydrogel formulations

After preparation and crosslinking of the hydrogel films, the samples were washed briefly with UPW. In the following, the samples underwent uniaxial compression testing using the Instron 5967 universal testing system (Instron, Germany) employing a 100 N loading cell. The hydrogel

specimens were compressed at a rate of 1 mm/min until a maximum strain equivalent to 20%. Subsequently, the effective stiffness was determined from the stress-strain data in a strain range between 5 % to 10 %. The measurements were performed with six replicates (n = 6).

Bioactivity study

For studying the bioactivity of BG-containing ADA-GEL hydrogels, a bioactivity study was performed under sterile conditions by immersing sterile hydrogel films in sterile filtrated simulated body fluid (SBF) in a shaking incubator set to 37°C and 80 rpm. The SBF was prepared following the protocol by Kokubo *et al.* [5] and each film was immersed in 25 mL of SBF [5]. The SBF was exchanged twice a week to maintain its ionic composition. After 14 days of incubation, the hydrogel films were removed from the SBF solution and rinsed briefly with UPW. Subsequently, the samples were freeze-dried to investigate potentially formed hydroxyapatite-like layers by X-ray diffraction (XRD, MiniFlex 600, Rigaku) or subjected to an ethanol washing series and critical point drying for an investigation by scanning electron microscopy (SEM, Carl Zeiss GmbH, Germany).

7. Characterization of hydrogel coating on gyroid scaffolds

Morphology of coated gyroid scaffolds

To investigate the feasibility of coating porous Ti-6Al-4V scaffolds, the scaffolds were dip-coated with ADA-GEL hydrogels as described before and subsequently cut in half by spark erosion to investigate whether the inside of gyroid scaffolds of different pore sizes can be coated with the selected hydrogel formulations. The cut hydrogel-coated gyroids were investigated by light microscopy (Stemi 508, Carl Zeiss GmbH, Germany).

Adhesion strength by pull-off test and cross hatch method

The evaluation of the adhesion of different hydrogel coatings to titanium alloy plates was conducted through pull-off testing and the cross hatch test. For all adhesion strength tests, the titanium alloy plates were first coated with the respective hydrogel formulation and subsequently freeze-dried. The pull-up test quantified the perpendicular force required to detach a specified area of the coating from the titanium alloy substrate. In this test, a loading fixture (d = 10 mm) was attached to the coating by glue. An actuator (PosiTest AT-A Automatic Adhesion Tester, DeFelsko) was then affixed to the dolly, applying a controlled and gradual pull-off pressure with a pull-off pressure rate of 0.1 MPa/s. The pull-up pressure was measured in triplicates (n = 3).

On the other hand, the tape test served as a qualitative assessment of the degree of adhesion of the coating to the titanium alloy substrate. To achieve this, a cutting blade (Elcometer® 107 Cross Hatch Cutter) was used to create a lattice pattern on the freeze-dried coating, scratching it both horizontally and vertically. Subsequently, an ISO adhesive tape was applied over the lattice pattern

and removed promptly. Based on the appearance of the coating after tape removal, the results were assessed following ASTM D3359-23 classifications.

8. *In vitro* testing

For investigating the cell-material interactions, the osteosarcoma cell line MG-63 was used. The cells were expanded in T-75 flasks using a maintenance medium composed of DMEM (1g/L D-glucose) supplemented with 10 v/v% fetal bovine serum (FBS) and 1 v/v% penicillin/streptomycin (PS) and kept in an incubator at 37°C, 5% CO_2 and 95% relative humidity. On the day of seeding, the cells were trypsinized and counted using a Neubauer counting chamber. In the following, 50.000 cells were suspended in 100 µL of medium and seeded on hydrogel-coated gyroids (UC4, pore size of 1.15 mm). The samples were then transferred to the incubator for 30 min to facilitate initial cell attachment and subsequently fresh medium was added for providing all needed nutrients. During the incubation period, the medium was exchanged three times a week.

To assess the metabolic activity of seeded cells at 1 day and 7 days post-seeding, a WST-8 viability assay (Cell Counting Kit-8, Sigma Aldrich) was employed. Initially, the cell-seeded samples were transferred to a new wellplate to eliminate any potential influence from cells grown on tissue culture polystyrene (TCPS). Subsequently, the samples (n = 3) were incubated in a 3 v/v% WST-8 solution in the respective cell culture medium (CCM) for a duration of 3 hours at 37°C, 5% CO_2, and 95% relative humidity. Following incubation, 100 µL of supernatant from each sample was transferred to a 96-well plate (Sarstedt, Germany) in triplicates, and the absorbance at 450 nm was measured using a plate reader (Phomo, Anthos Mikrosysteme GmbH, Krefeld, Germany).

To evaluate the cell morphology, the seeded cells were stained with Rhodamine-Phalloidin, and DAPI. After the WST-8 assay, the cells were briefly washed with HBSS and fixed using a 4 w/v% formaldehyde solution for 15 min. The next step involved cell permeabilization for 5 minutes with 0.1% TritonX-100, followed by incubation in 8 µL/mL Rhodamine-Phalloidin in HBSS for 1 hour at room temperature. Finally, the cells were stained with 1 µL/mL DAPI in HBSS for 30 minutes and examined using fluorescence microscopy (AXIO Observer.D1, Carl Zeiss Microscopy, Germany).

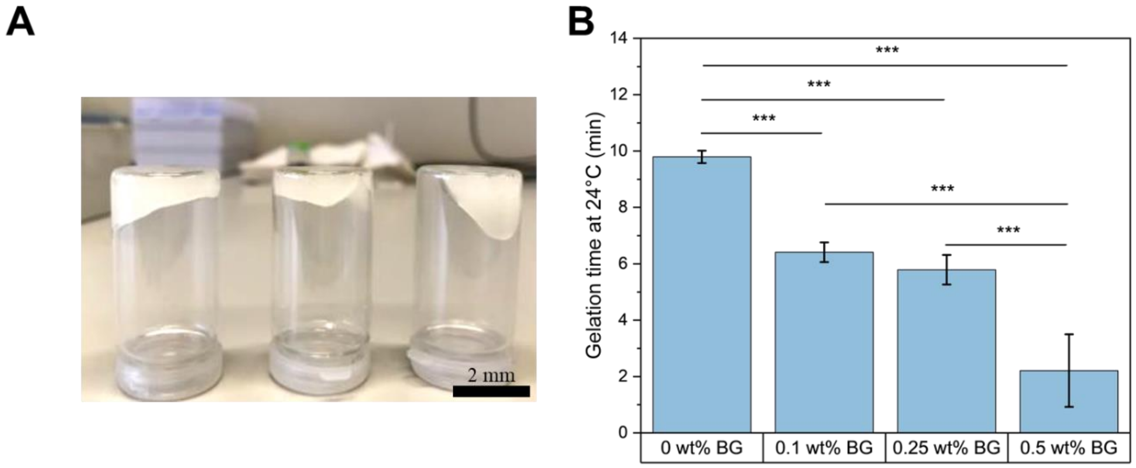

Figure S1. Investigation of the gelation time of 2.5-5.0 w/v% ADA-GEL hydrogels containing different concentrations of 45S5 Bioglass at room temperature for the initial decision on hydrogel formulations showing a finished tube inversion test of ADA-GEL-0.5BG (A) and the respective plot of determined gelation times (B).

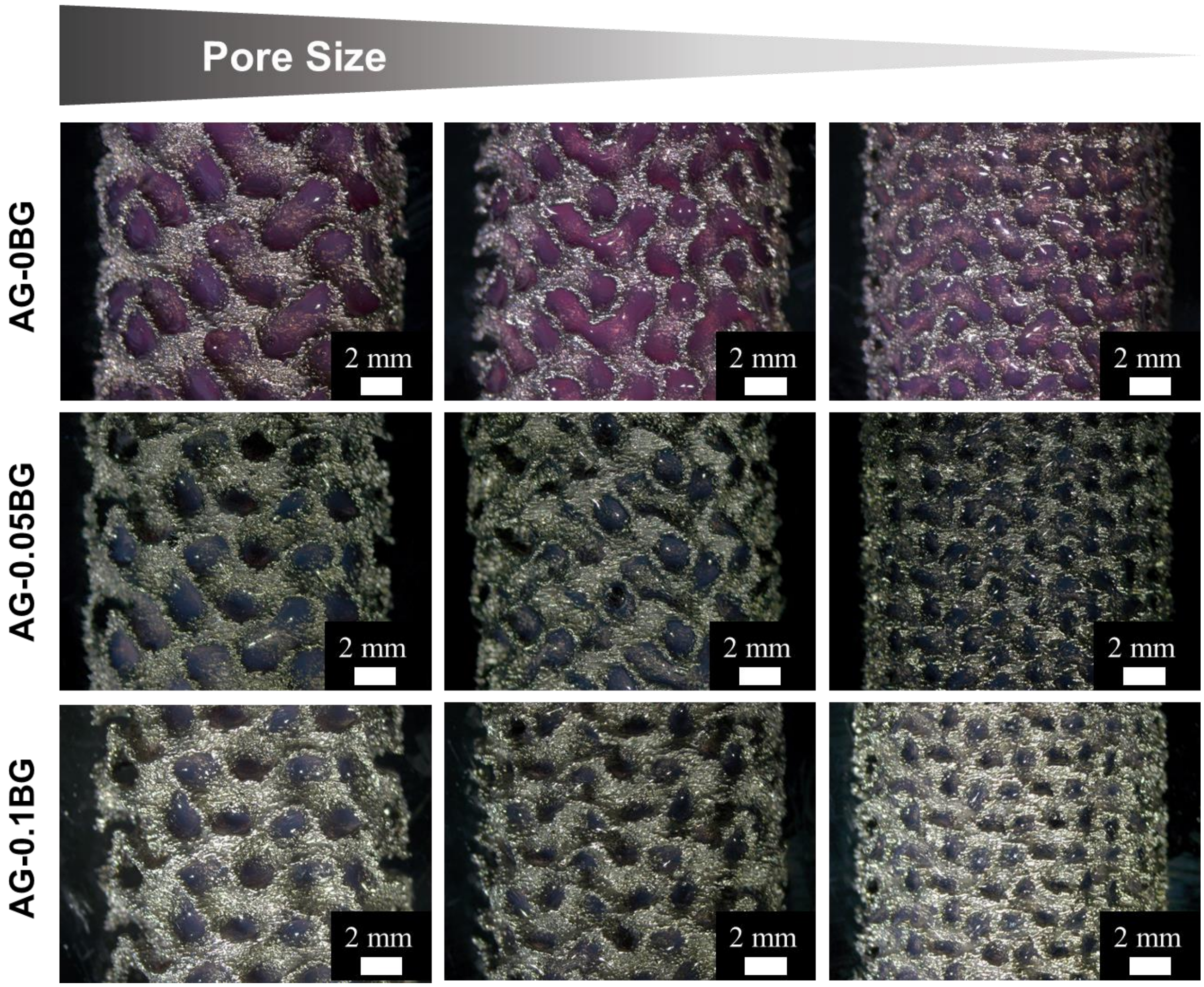

Figure S2. Light microscopy images of ADA-GEL-BG coated Ti-6Al-4V gyroid scaffolds from the outside. In order to visualize the coating more readily, red food coloring was added to the hydrogels.

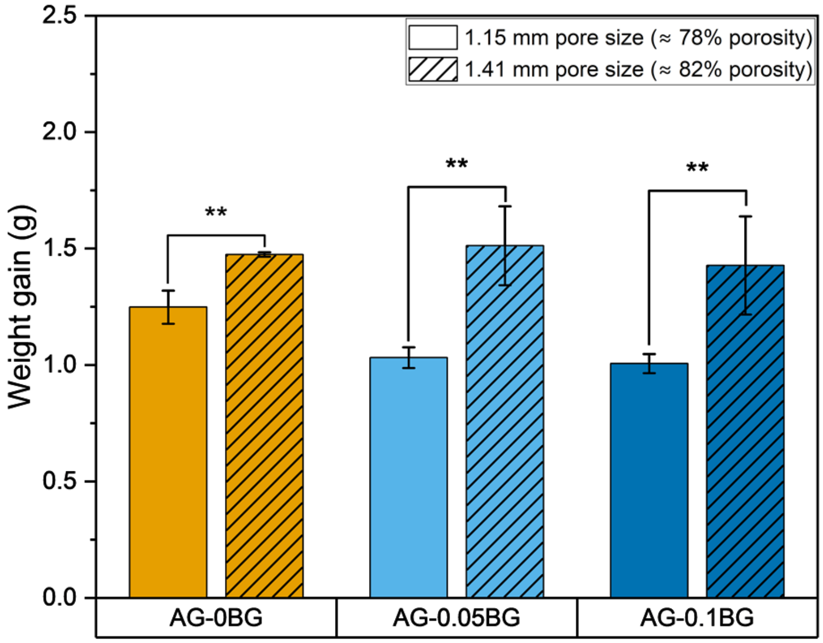

Figure S3: Results of the investigation of weight gain of hydrogel-coated gyroid scaffolds of different pore sizes indicating a significant increase in gained weight with larger pore sizes due to a larger space for hydrogel penetration and reduced capillary effects.

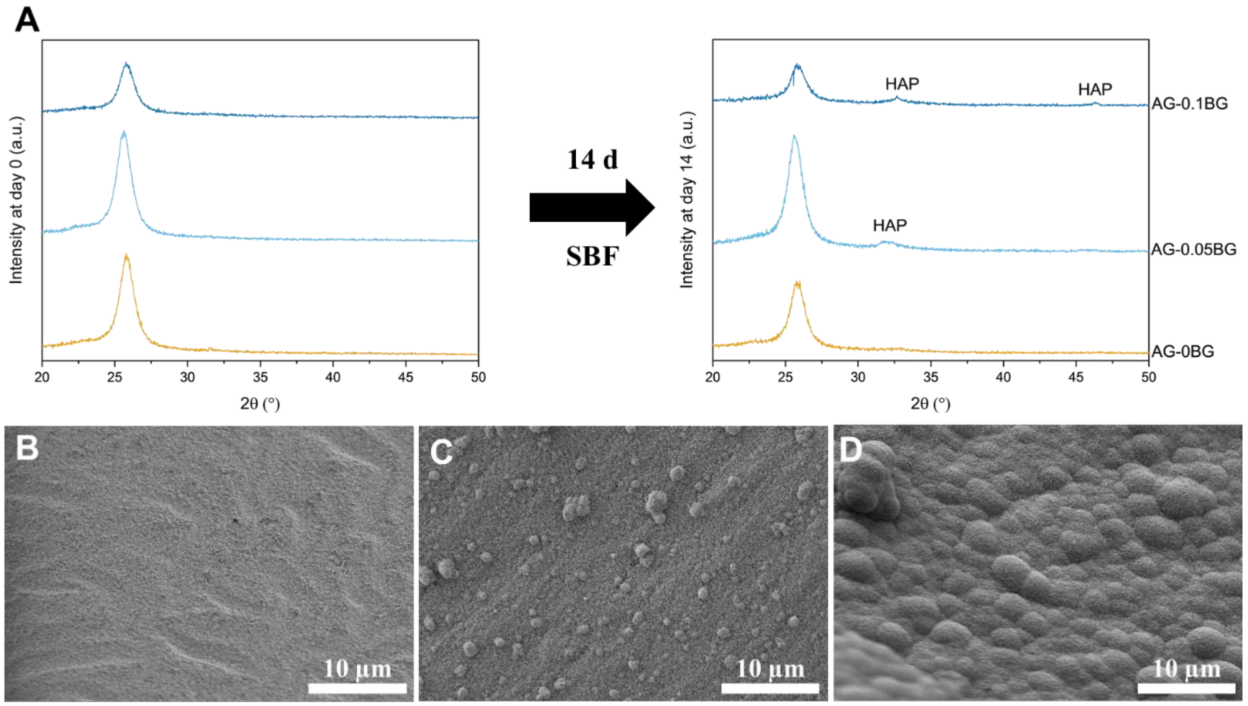

Figure S4. Results of the bioactivity study showing the XRD spectra of ADA-GEL-based hydrogels at day 0 and after 14 days of incubation in SBF with peaks at 32° (211) and 46° (222) assigned to apatite according to the JCPDS standards[6] (A). The SEM images show the hydrogel surface after 14 days incubation in SBF solution with ADA-GEL (B) showing a relatively smooth surface compared to the surface of ADA-GEL-0.05BG (C) and ADA-GEL-0.1BG (D) where the typical cauliflower-like calcium-phosphate agglomerates caused by the incorporation of 45S5 Bioglass can be observed.

References

(1) Polley, C.; Radlof, W.; Hauschulz, F.; Benz, C.; Sander, M.; Seitz, H. Morphological and Mechanical Characterisation of Three-Dimensional Gyroid Structures Fabricated by Electron Beam Melting for the Use as a Porous Biomaterial. *J Mech Behav Biomed Mater* **2022**, *125*. https://doi.org/10.1016/j.jmbbm.2021.104882.

(2) Karakaya, E.; Schöbel, L.; Zhong, Y.; Hazur, J.; Heid, S.; Forster, L.; Teßmar, J.; Boccaccini, A. R.; Detsch, R. How to Determine a Suitable Alginate for Biofabrication Approaches Using an Extensive Alginate Library? *Biomacromolecules* **2023**, *24* (7), 2982–2997. https://doi.org/10.1021/acs.biomac.2c01282.

(3) Reakasame, S.; Jin, A.; Zheng, K.; Qu, M.; Boccaccini, A. R. Biofabrication and Characterization of Alginate Dialdehyde-Gelatin Microcapsules Incorporating Bioactive Glass for Cell Delivery Application. *Macromol Biosci* **2020**, *20* (10). https://doi.org/10.1002/mabi.202000138.

(4) Nguyen, T. P.; Lee, B. T. Fabrication of Oxidized Alginate-Gelatin-BCP Hydrogels and Evaluation of the Microstructure, Material Properties and Biocompatibility for Bone Tissue Regeneration. *J Biomater Appl* **2012**, *27* (3), 311–321. https://doi.org/10.1177/0885328211404265.

(5) Kokubo, T.; Takadama, H. How Useful Is SBF in Predicting in Vivo Bone Bioactivity? *Biomaterials* **2006**, *27* (15), 2907–2915. https://doi.org/10.1016/j.biomaterials.2006.01.017.

(6) Rezaei, Y.; Moztarzadeh, F.; Shahabi, S.; Tahriri, M. Synthesis, Characterization, and in Vitro Bioactivity of Sol-Gel-Derived SiO2-CaO-P2O5-MgO-SrO Bioactive Glass. *Synthesis and Reactivity in Inorganic, Metal-Organic and Nano-Metal Chemistry* **2014**, *44* (5), 692–701. https://doi.org/10.1080/15533174.2013.783869.
